# METTL9-SLC7A11 axis promotes hepatocellular carcinoma progression through ferroptosis inhibition

**DOI:** 10.1038/s41420-023-01723-4

**Published:** 2023-11-28

**Authors:** Fangfang Bi, Yuxiong Qiu, Zongfeng Wu, Shaoru Liu, Dinglan Zuo, Zhenkun Huang, Binkui Li, Yunfei Yuan, Yi Niu, Jiliang Qiu

**Affiliations:** 1 State Key Laboratory of Oncology in South China and Collaborative Innovation Center for Cancer Medicine, Sun Yat-sen University Cancer Center, Guangzhou, China; 2 Department of Liver Surgery, Sun Yat-sen University Cancer Center, Guangzhou, China

**Keywords:** Oncogenes, Cell invasion

## Abstract

Methytransferase-like proteins 9 (METTL9) has been characterized as an oncogene in several cancers, however, its role in hepatocellular carcinoma (HCC) remains unknown. Here, we investigated the function and molecular mechanism of METTL9 in HCC. We showed that METTL9 expression was elevated in HCC, and its high expression was associated with poor survival outcomes. Knockdown of METTL9 observed a significant inhibition of HCC cell viability, migration, and invasion both in vitro and in vivo. By contrast, METTL9 overexpression HCC cells obtained stronger abilities in cell proliferation and migration. Mechanistically, we discovered that METTL9 knockdown led to a reduction in the expression level of SLC7A11, a key suppressor of ferroptosis, in turn, promoted ferroptosis in HCC cells, impeding the progression of HCC. Moreover, we have proved that targeting METTL9 could significantly restrain the growth of HCC patient-derived xenograft (PDX). Our study established METTL9 as a critical role in promoting HCC development and provides a foundation for further investigation and potential therapeutic interventions targeting ferroptosis in HCC.

## Introduction

Hepatocellular carcinoma (HCC) is ranked as the sixth most frequently diagnosed cancer worldwide and the third most common cause of cancer-related death [1, 2]. Early-stage HCC may be curable by resection, liver transplantation, or ablation, however, most patients present with unresectable disease, resulting in a relative 5-year survival rate of approximately 18% [3, 4]. In recent year, although remarkable progress has been made in the treatment of HCC, such as loco-regional therapy, targeted drug, and immunotherapy [3, 5], the survival was still unsatisfied. Hence, it’s of great importance to investigate the mechanism of HCC occurrence and development and explore effective targets for HCC treatment.

Methytransferase-like proteins (METTL) are part of a large protein family which is characterized by the presence of binding domains for S-adenosylmethionine [6]. Previous studies reported that METTL family genes are involved in diverse cellular processes and cancer progression [7–12]. METTL9, a member of the METTL family, has been recently characterized as a novel N1-histidine methyltransferase [13, 14]. It was reported that METTL9 could catalyze N1-methylhistidine formation in the proinflammatory protein S100A9 [15], and our previous study demonstrated that S100A9 promoted the growth and metastatic ability of HCC [16]. Moreover, METTL9 was essential for tumor growth by regulating zine transport proteins inducing ER stress in prostate cancer [14]. Knockdown of METTL9 significantly inhibited migration and invasion and reduced mitochondrial Complex I activity in human scirrhous gastric cancer [17]. Although METTL9 has been characterized as an oncogene in several cancers, its biological function in HCC is poorly understood and the knowledge of underlying molecular mechanism is limited, and remains to be elucidated.

Ferroptosis, a newly identified form of regulated cell death, is characterized by iron-dependent accumulation of lipid peroxidation, and differs from apoptosis, unregulating necrosis, and necroptosis [18–21]. Growing evidence supports that ferroptosis has emerged as a potential therapeutic target for anticancer therapy [22, 23]. Glutathione, synthesized from cysteine, glutamate, and glycine, stands as the most prevalent antioxidant within cells, among which cysteine serves as the rate-limiting precursor of glutathione. A significant number of cancer cells mainly rely on the cystine transporter system Xc^-^ to facilitate the import of cystine which is subsequently transformed into cysteine in the cytosol. System Xc^-^ is an amino acid antiporter that exports intracellular glutamate and import extracellular cystine [24], which plays a critical role in ferroptosis. The solute carrier family 7 member 11 (SLC7A11) is the light chain subunit of system Xc^-^, mediates the cystine/glutamate antiporter activity in the system Xc^-^, functions to import cystine for glutathione biosynthesis and antioxidant defense, playing a role in negatively regulating ferroptosis [25]. Additionally, an increasing number of studies have confirmed that SLC7A11 inhibits ferroptosis in tumor cells [26–28]. Nevertheless, it remains obscure whether SLC7A11 and ferroptosis subsequently influence the prognosis of HCC. Hence, exploring the regulatory mechanisms of SLC7A11 has been a focus of significant importance.

In the present study, we systematically showed that METTL9 was upregulated in HCC and high METTL9 expression levels were associated with poor prognosis. Down-regulation of METTL9 suppressed HCC cell proliferation and migration in vitro and in vivo, and vice versa. Mechanistically, we identified that METTL9 acted as a regulator of SLC7A11, increased the expression of SLC7A11, leading to inhibition of cell ferroptosis, subsequently inducing the growth and metastasis of HCC. Targeting METTL9 could obviously curb the growth of HCC PDX. To the best of our knowledge, this is the first time to elucidate the effect of METTL9 on HCC and the correlation of METTL9 with ferroptosis. Our findings uncovered METTL9 as a new promising target of HCC treatment and give an implication for understanding the mechanism of ferroptosis pathway in HCC.

## Results

### METTL9 is upregulated in HCC and correlated with poor prognosis

**Fig. 1:**
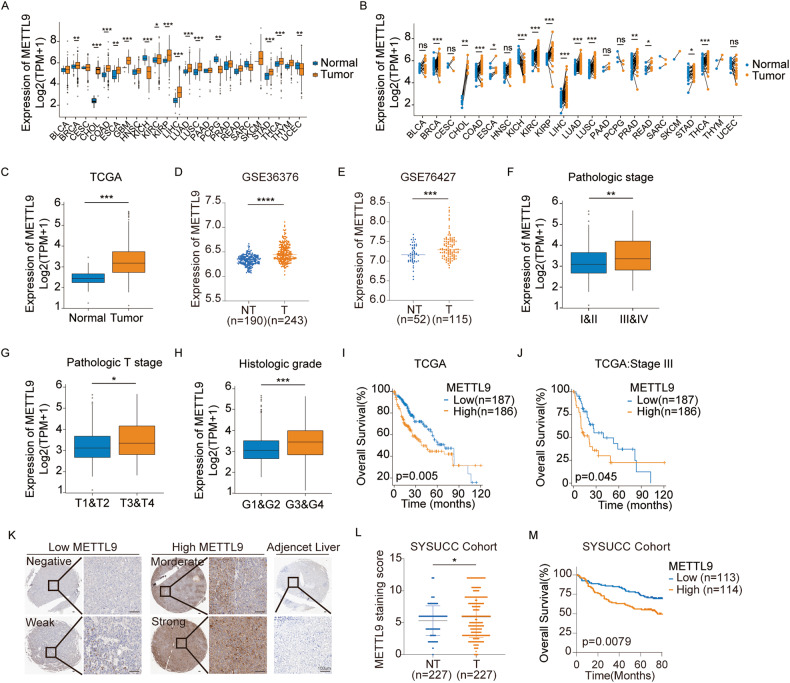
METTL9 is upregulated in HCC and correlated with poor survival. **A** METTL9 mRNA expression between normal and tumor tissues in 24 cancer types in TCGA datasets. **B** METTL9 mRNA expression in paired tissues in 23 cancer types in TCGA datasets. **C**–**E** METTL9 mRNA expression level in TCGA-LIHC normal and tumor tissues (**C**), GSE36376 (**D**), GSE76427 (**E**). **F**–**H** METTL9 mRNA expression level in clinical pathologic stage (**F**), pathologic T stage (**G**) and histologic grade (**H**) in TCGA-LIHC dataset. **I**, **J** Kaplan–Meier overall survival curves of individuals (**I**) and pathologic stage III (**J**) patients with high and low METTL9 expression in TCGA-LIHC dataset. **K** IHC analysis of METTL9 in HCC tissue microarray chip. The representative images of different levels of METTL9 expression in HCC (left) and adjacent non-tumor liver tissues (right) are presented. Scale bar,100 μm. **L** Quantification of METTL9 expression in HCC and adjacent non-tumor tissues according to IHC scores in SYSUCC cohort (*n* = 227). Relative IHC scores are shown as mean ± SD. **M**. Kaplan–Meier overall survival curves of patients with high and low METTL9 expression in SYSUCC cohort.

To elucidate the potential role of METTL9 in cancers, we initially investigated the expression levels of METTL9 in tumors using The Cancer Genome Atlas (TCGA) dataset. As shown in Fig. 1A–C, METTL9 expression levels were generally upregulated in tumor tissues, including HCC. GEO datasets (GSE36376 and GSE76427) also demonstrated significantly higher METTL9 expression in tumor tissues compared to non-tumor tissues (Fig. 1D, E). Further analysis of the TCGA-LIHC data showed that METTL9 expression was significantly higher in pathological TNM stages III & IV compared to stages I & II, in T3 & T4 stages compared to T1 & T2 stages, and in G3 & G4 tissue grades compared to G1 & G2 tissue grades respectively (Fig. 1F–H), which indicated that the expression level of METTL9 increased with the advanced stage and the higher malignancy of HCC. Next, the results of Kaplan–Meier survival analysis of TCGA-LIHC data showed that high METTL9 expression levels were associated with relatively shorter overall survival (Fig. 1I, J).

To further validate the results of TCGA data, we performed immunohistochemistry (IHC) staining on liver cancer tissue microarray at Sun Yat-Sen University Cancer Center (SYSUCC) and assessed the relationship between METTL9 expression level and patient survival prognosis. Our data revealed significant upregulation of METTL9 expression in liver cancer tissues compared to non-cancerous tissues (Fig. 1K, L), Additionally, patients with high METTL9 expression showed a significantly shorter overall survival (Fig. 1M). Overall, these results provide compelling evidence that METTL9 expression is upregulated and correlates with poor prognosis in HCC.

### Knockdown of METTL9 inhibits HCC progression in vitro and in vivo

**Fig. 2:**
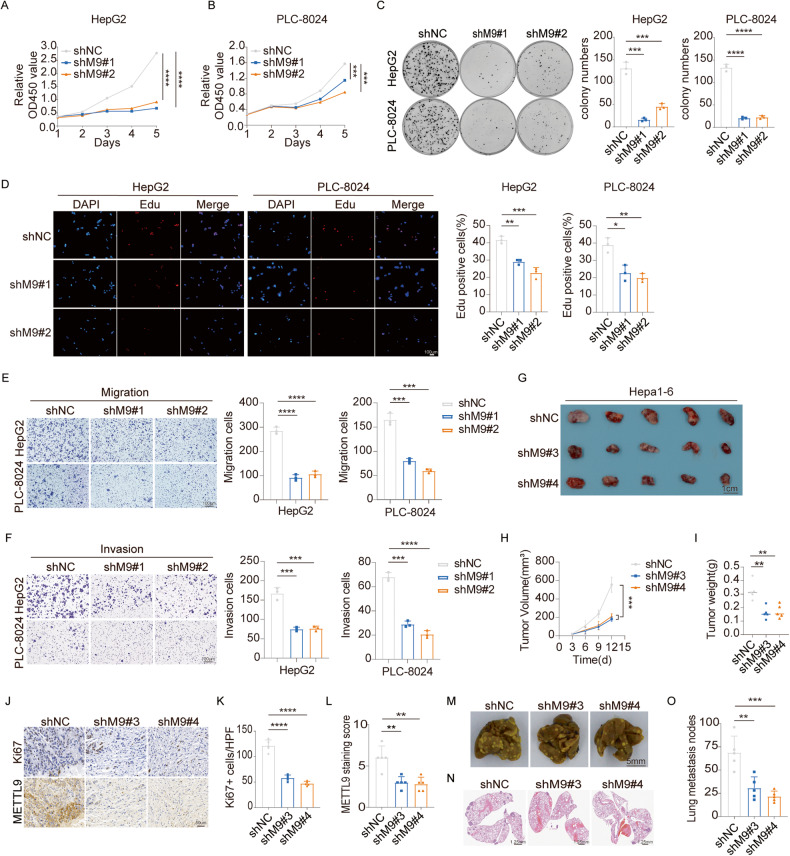
Knockdown of METTL9 inhibits HCC progression in vitro and in vivo. **A**, **B** Knockdown of METTL9 reduced cell proliferation of HepG2 and PLC-8024 cells, as evaluated by CCK-8 assay. **C** Knockdown of METTL9 impaired colony formation of HepG2 and PLC-8024 cells. *n* = 3 independent experiments. **D** Knockdown of METTL9 significantly reduced HepG2 and PLC-8024 cell proliferation analyzed by Edu cell proliferation assay. Edu positive rates are expressed as mean ± SD. *n* = 3 independent experiments. Scale bar,100 μm. **E**, **F** Knockdown of METTL9 significantly decreased migration and invasion of HepG2 and PLC-8024 cells by transwell assay. Quantifications of migrating and invading cells are shown as mean ± SD. *n* = 3 independent experiments. Scale bar,100 μm. **G** Knockdown of METTL9 significantly suppressed tumor growth in subcutaneous xenograft mouse models. Representative images of xenograft tumors induced by indicated cells in NSG mice. Scale bar,1 cm. **H** Tumor size was measured starting at 3 days after inoculation. **I** Statistical analysis of tumor weights in each group was expressed as median(*n* = 5). **J** The level of Ki67 and METTL9 in the indicated xenograft tumors by IHC assay. Scale bar, 50 μm. **K**, **L** Quantification of Ki67+ stained cells and METTL9 staining score in tumors (*n* = 5). **M** Knockdown of METTL9 suppressed the lung metastatic capacity of HCC cells in NSG mice. Scale bar, 0.5 cm. **N** Representative images of lung metastasis node HE staining. Scale bar, 1.25 mm. **O** Statistical analysis of lung metastasis nodes in each group was expressed as mean ± SD (*n* = 5).

To investigate the potential oncogenic role of METTL9 in HCC, we first assessed the mRNA and protein expression levels of METTL9 in various HCC cell lines (HepG2, PLC-8024, Hep3B, Huh7, LM3, and MHCC97H) and normal hepatocytes (MIHA). The results revealed relatively higher expression levels of METTL9 in HepG2 and PLC-8024 cells compared to MIHA cells (Fig S1A, B). Thus, we established stable knockdown METTL9 expression using two short hairpin RNAs-mediated gene silencing (shM9#1 and shM9#2) in HepG2 and PLC-8024 cell lines for loss-of-function experiments (Fig. S1C-D). CCK-8 cell proliferation assay, clone formation and EDU proliferation assay were conducted to investigate the function of METTL9 on HCC cells. Our results showed that the knockdown of METTL9 significantly inhibited cell viability (Fig. 2A, B), decreased in the number of cell colonies (Fig. 2C) and reduced the proliferation rate in HCC cells (Fig. 2D). Additionally, to evaluate the impact of METTL9 on cell migration and invasion, we performed transwell migration and invasion assays, which revealed that knockdown of METTL9 significantly suppressed the migration and invasion abilities of both HepG2 and PLC-8024 cell lines (Fig. 2E, F). Next, using two short hairpin RNAs (sh#3 and sh#4), We construct METTL9 stable knockdown murine-derived Hepa1-6 cells (Fig S1E, F). The genetically engineered Hepa1-6 cells were subcutaneously injected in NSG mice. Tumor growths were monitored from 3 days after injection. Knockdown of METTL9 could obviously decelerate HCC tumor growth (Fig. 2G, H). 12 days after inoculation, tumors were excised and weighted. Compared to the control group, the tumor weights were significantly lighter in the METTL9 knockdown group (Fig. 2I). IHC staining showed that the control HCC tissues had a higher proportion of Ki67-positive cells and METTL9 expression levels than the METTL9 knockdown group (Fig. 2J–L). Furthermore, we proceeded to construct a lung metastatic tumor model by tail vein injection of NSG mice with the genetically engineered Hepa1-6 cells to further evaluate the metastatic ability in vivo. As expected, the silencing of METTL9 significantly reduced the number of lung metastatic nodes compared to the control group (Fig. 2M–O).

### Elevated METTL9 expression promotes HCC progression in vitro and in vivo

**Fig. 3:**
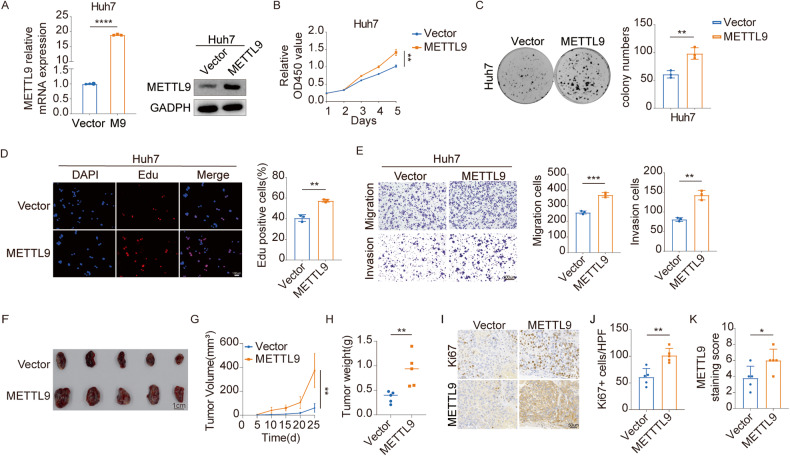
Elevated METTL9 expression promotes HCC progression in vitro and in vivo. **A** Efficiency of METTL9 stable overexpression was verified in Huh7 cell line by real-time PCR and Western blot. **B** Overexpression of METTL9 promotes cell proliferation of Huh7 cells, as evaluated by CCK-8 assay. **C** Overexpression of METTL9 promotes colony formation of Huh7 cells. *n* = 3 independent experiments. **D** Overexpression of METTL9 significantly increased Huh7 cell proliferation analyzed by Edu cell proliferation assay. Edu positive rates are expressed as mean ± SD. *n* = 3 independent experiments. Scale bar,100 μm. **E** METTL9 overexpression significantly increased migration and invasion of Huh7 cells by transwell assay. Quantifications of migrating and invading cells are shown as mean ± SD. *n* = 3 independent experiments. Scale bar,100 μm. **F** Overexpression of METTL9 significantly promotes tumor growth in subcutaneous xenograft mouse models. Representative images of xenograft tumors induced by indicated cells in NSG mice. Scale bar,1 cm **G** Tumor size was measured starting at 5 days after inoculation. **H** Statistical analysis of tumor weights in each group were expressed as median (*n* = 5). **I** The level of Ki67 and METTL9 in the indicated xenograft tumors by IHC assay. Scale bar, 50 μm. **J**, **K** Quantification of Ki67+ stained cells and METTL9 staining score in tumors (*n* = 5).

To further elucidate the biological function of METTL9 in HCC, we conducted gain-of-function assays by overexpressing METTL9 in Huh7 cells (Fig. 3A). CCK-8 cell proliferation assay, colony formation, and Edu proliferation assay were performed to assess the impact of METTL9 overexpression on HCC cells. The results revealed that overexpression of METTL9 significantly enhanced cell viability, clonogenic ability, and proliferation of Huh7 cells compared to the vector group (Fig. 3B–D). In line with our previous findings, transwell assays demonstrated that the number of migrated and invaded cells in the METTL9 overexpression group was significantly higher than that in the Ctrl (Fig. 3E), indicating that METTL9 up-regulation promoted the migration and invasion abilities in HCC cells.

In vivo, we injected METTL9 overexpression Huh7 cells and the control vector cells into NSG mice. Tumor growth was monitored for 5 days after injection. It was found that METTL9 overexpression led to greater proliferation capacity, larger tumor size, and heavier tumor weights compared to the vector group (Fig. 3F–H). Consistently, IHC staining showed relatively higher METTL9 staining scores and a higher proportion of Ki67 positive cells in the METTL9 overexpression group (Fig. 3I–K). Taken together, our data provided strong evidence supporting the oncogenic function of METTL9 in promoting the progression of HCC both in vitro and in vivo.

### METTL9 affects HCC progression by regulating ferroptosis

**Fig. 4:**
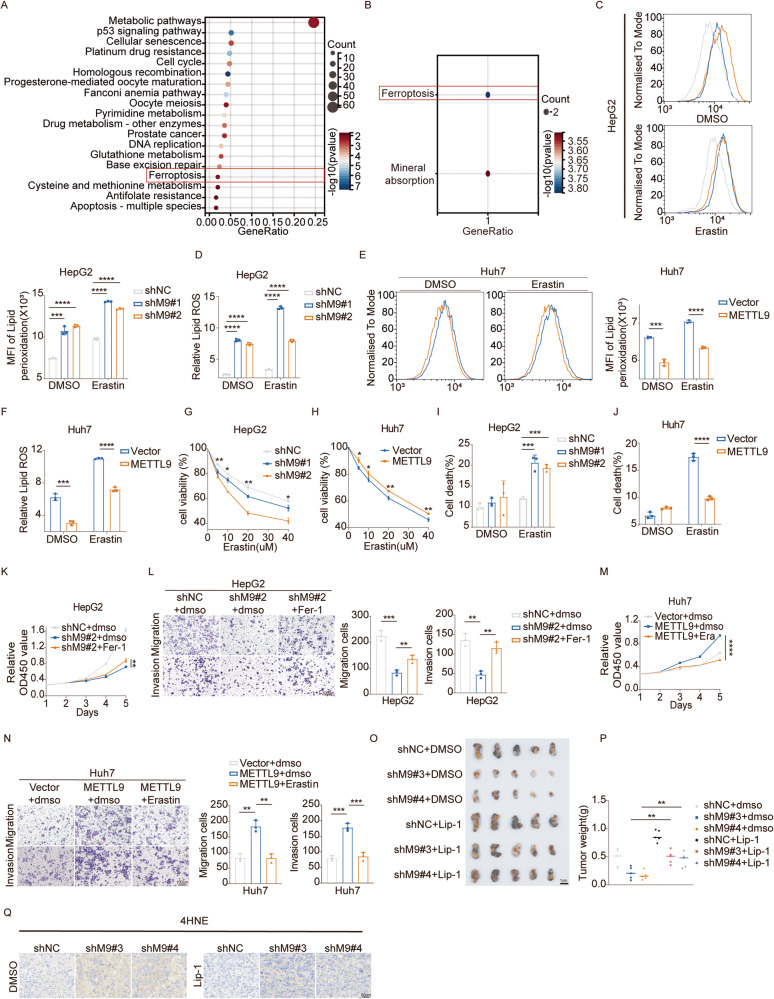
METTL9 affects HCC progression by regulating ferroptosis. **A** KEGG pathway enrichment analysis of DEGs by shMETTL9 and shNC in HepG2 cells. **B** KEGG pathway enrichment analysis of SLC30A, SLC39A genes families. **C**–**F** 24 h after DMSO or 10 μm Erastin treatment, lipid peroxidation and lipid ROS production were respectively assessed by Liperfluo and BDP 581/591 C11 staining in HepG2 stably expressing control (shNC) or METTL9 knockdown (sh#1, sh#2) cells and Huh7 stably expressing vector or METTL9 overexpression(METTL9) cells followed by flow cytometry. *n* = 3 independent experiments. **G**, **H** Cell viability of HepG2 knockdown or Huh7 overexpression METTL9 cells was determined after treatment with different concentrations of Erastin for 36 h by CCK-8 assay. *n* = 3 independent experiments. **I**, **J** Cell death of HepG2 knockdown or Huh7 overexpression METTL9 cells after treatment with 10 μM Erastin or DMSO for 24 h. *n* = 3 independent experiments. **K**, **L** CCK8 and transwell assays in HepG2 shMETTL9#2 and shNC cells with DMSO or 2 μM Fer-1. (L)Scale bar,100 μm. **M**, **N** CCK8 and transwell assays in Huh7 METTL9 overexpression cells with DMSO or 10 μM Erastin. (N)Scale bar,100 μm. **O** Different indicated Hepa1-6 cells were injected subcutaneously into NSG mice. Lip-1 (10 mg/kg) or DMSO was injected intraperitoneally every other day starting at 5 days after inoculation (*n* = 5 per group). Scale bar,1 cm. **P** Statistical analysis of tumor weights in each group was expressed as median (*n* = 5). **Q** Representative images of IHC staining of 4HNE in xenografts. Scale bar, 50 μm.

To elucidate the potential molecular mechanism underlying METTL9-mediated HCC progression. Proteomic sequencing on shNC and shM9#1 HepG2 cell had been conducted to identify differentially expressed genes (DEGs) (|log_2_FC| >1, *p* < 0.05). 590 DEGs from the sequencing results were subjected to Kyoto Encyclopedia of Genes and Genomes (KEGG) pathway enrichment analysis, revealing the top 20 signaling pathways (Fig. 4A). SLC39A and SLC30A family genes were reported as the main substrates regulated by METTL9 [14], Thus, we performed KEGG pathway enrichment analysis on these two family genes (Fig. 4B). For the ferroptosis signaling pathway appeared in both sets of enrichment analysis results, we hypothesized that ferroptosis might play a key role in METTL9 regulation in HCC. To verify our hypothesis, lipid peroxidation and lipid ROS levels which are closely associated with ferroptosis were measured using flow cytometry in vitro. The results showed that the mean fluorescence intensity (MFI) of lipid peroxidation and relative lipid ROS levels were significantly increased in METTL9 knockdown HepG2 and PLC-8024 cells. Moreover, these effects were even more pronounced with the addition of Erastin, a ferroptosis inducer (Fig. 4C, D, Fig S2B, C). Conversely, overexpression of METTL9 in Huh7 cells suppressed MFI of lipid peroxidation and relative lipid ROS levels compared to the vector group(Fig. 4E, F). Further, we have shown that silencing of METTL9 significantly increased Erastin-induced growth inhibition (Fig. 4G, Fig S2D) and cell death(Fig. 4I) in HCC cells, while overexpression of METTL9 decreased Erastin-induced growth inhibition and cell death in Huh7 cells (Figs. 4H, J). Our data tentatively suggest that METTL9 participated in the regulation of ferroptosis in HCC.

Next, to investigate whether METTL9 induced HCC progression via mediating ferroptosis, we treated METTL9 knockdown cells with ferroptosis inhibitor ferrostatin-1 (Fer-1) and METTL9 overexpression cells with Erastin in vitro. CCK-8 proliferation and transwell assays indicated that the inhibiting effect of METTL9 knockdown on cell viability, migration, and invasion capacity in HepG2 and PLC-8024 cells could be rescued by Fer-1 (Fig. 4K, L, Fig S2E, F). Conversely, Erastin could curb METTL9 overexpression-induced Huh7 cells proliferation, migration, and invasion (Fig. 4M, N). Next, we further examined the effect of ferroptosis inhibitor Liproxstatin-1 (Lip-1) administration on tumor growth in vivo experiments. The results showed that Lip-1 partially restored tumor size and weight of METTL9 knockdown group (Fig. 4O, P). 4HNE staining was performed to assess lipid peroxidation levels in the xenografts. It was found that 4HNE levels were significantly higher in the METTL9 knockdown group. Lip-1 partially restored 4HNE level in the METTL9 knockdown group (Fig. 4Q). Collectively, these findings indicated that METTL9 mediated ferroptosis to regulate HCC progression.

### SLC7A11 is a major downstream player in METTL9 mediated ferroptosis in HCC

**Fig. 5:**
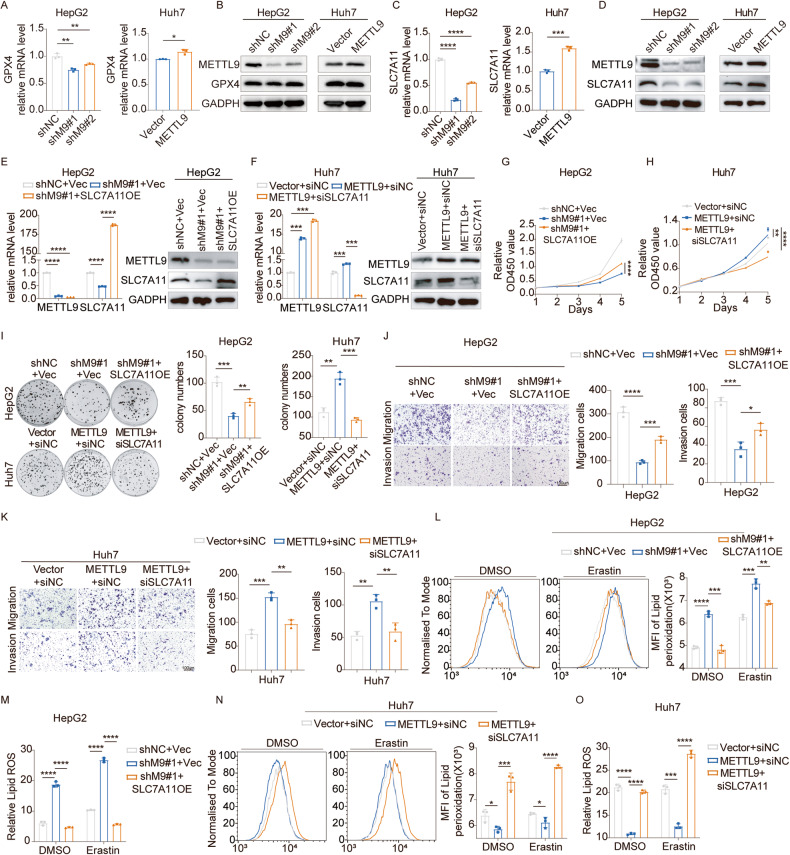
SLC7A11 is a major downstream player in METTL9-mediated ferroptosis in HCC. **A**, **B** GPX4 expression at the mRNA and protein levels in METTL9 stable knockdown HepG2 cell line and stable overexpression Huh7 cell line by real-time PCR (*n* = 3 independent experiments) and Western blot. **C**, **D** SLC7A11 relative mRNA and protein level was down-regulated in METTL9 stable knockdown HepG2 cell line and up-regulated in stable overexpression Huh7 cell line by real-time PCR (*n* = 3 independent experiments) and Western blot. **E**, **F** Efficiency verification in HepG2 cells stably expressing shMETTL9#1 with further overexpression SLC7A11 and Huh7 cells stably expressing METTL9 with further knockdown SLC7A11 by real-time PCR (*n* = 3 independent experiments) and Western blot. **G**–**K** Cell proliferation by CCK8 assay (**G**, **H**) and colony formation (**I**) and migration and invasion assay (**J**, **K**) was partially rescued in HepG2 shMETTL9#1 cells with further overexpression SLC7A11 and Huh7 METTL9 overexpression cells with knockdown SLC7A11. *n* = 3 independent experiments. **J**, **K** Scale bar,100 μm. **L**–**O** Lipid peroxidation (**L**, **N**) and lipid ROS (**M**, **O**) were assessed and partially rescued in HepG2 shMETTL9#1 cells with further overexpression SLC7A11 and Huh7 METTL9 overexpression cells with knockdown SLC7A11. *n* = 3 independent experiments.

To further investigate the molecular mechanisms underlying ferroptosis regulated by METTL9, we analyzed the proteomic sequencing data of differentially expressed genes (DEGs) associated with ferroptosis [23]. Combined with numerous studies in the literature related to ferroptosis, we noted the SLC7A11 and Glutathione peroxidase 4 (GPX4), two of the key molecules related to ferroptosis [25, 29–31]. Then we found that GPX4 mRNA level only decreased by about 20% after the knockdown of METTL9 in HepG2 cell lines, and increased by 1.1-fold after overexpression of METTL9 in Huh7 cell lines compared to the control group, while its protein level did not change significantly in the knockdown or overexpression group compared to the control group (Fig. 5A, B), which was also consistent with our proteomics quantitative sequencing data. However, both the mRNA and protein levels of SLC7A11 were significantly down-regulated after the knockdown of METTL9 in HepG2 and PLC-8024 cell lines (Fig. 5C, D, Fig S4A), and up-regulated after overexpression of METTL9 in Huh7 cell line (Fig. 5C, D). Furthermore, TCGA-LIHC data showed a significant positive correlation between METTL9 and SLC7A11 (Fig S4B), confirming our RT-PCR results. As a result, we anchored the molecule SLC7A11 to continue the subsequent research.

We subsequently constructed siRNA to silence SLC7A11 in HCC cells (Fig S3A, B). Silencing of SLC7A11 resulted in a significant increase ferroptosis in HepG2 and PLC-8024 cells (Fig S3C–E). CCK-8 cell proliferation and clone formation assays revealed that inhibition of SLC7A11 expression restricted cell viability and proliferation capacity in HepG2 and PLC-8024 cells (Fig S3F–H). Transwell assays showed that migration and invasive capacity of HCC cells were significantly decreased in the siSLC7A11 group (Fig S3I, J). These results suggested that SLC7A11 acted as an inhibitor of ferroptosis and played an oncogenic role in promoting the progression of HCC. To validate that the effects of METTL9-mediated tumor promotion mainly depended on SLC7A11, We overexpressed SLC7A11 in stably METTL9-knockdown HepG2 and PLC-8024 cells, and knockdown SLC7A11 in stably METTL9-overexpressing Huh7 cells respectively (Fig. 5E, F, Fig S4C). A series of rescue experiments were then conducted. CCK-8 cell proliferation and colony formation assays revealed that overexpression of SLC7A11 partially rescued cell viability and growth inhibition caused by METTL9 knockdown in HepG2 and PLC-8024 cells (Fig. 5G, I, Fig S4D, E). Further, the knockdown of SLC7A11 partially rescued the growth-promoting ability in Huh7 cells overexpressing METTL9 (Fig. 5H, I). Similar results were obtained in migration and invasion assays (Fig. 5J, K, Fig S4F). Moreover, the lipid peroxidation and lipid ROS assays indicated that overexpression of SLC7A11 and knockdown METTL9 significantly downregulated the level of ferroptosis compared to the METTL9 knockdown group in HepG2 and PLC-8024 cells (Fig. 5L, M, Fig S4G, H). Similarly, stable expression of METTL9 and knockdown of SLC7A11 further promoted ferroptosis compared to overexpression of METTL9 in Huh7 cells (Fig. 5N, O). Taken together, our results suggest that METTL9 exerts a regulatory function in HCC by regulating the expression of SLC7A11 and thus affecting ferroptosis.

### Clinical significance of METTL9 inhibition in HCC

**Fig. 6:**
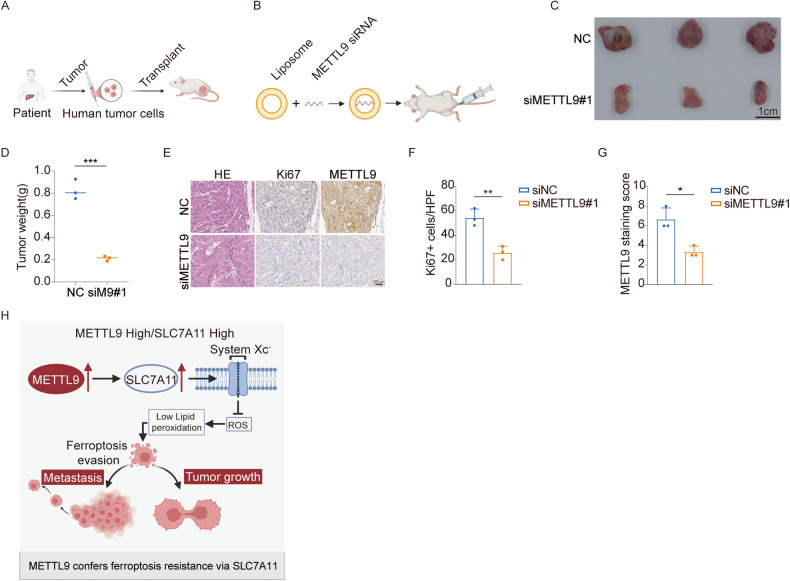
Clinical significance of METTL9 inhibition in HCC. **A** Schematic diagram of PDX model. **B** Schematic diagram showing targeting of METTL9 by siRNAs in vivo. **C** Inhibition of METTL9 by siRNA in HCC patient-derived xenograft (HCC-PDX). Scale bar, 1 cm. **D** Tumor weight of HCC-PDX treated with in vivo-optimized siRNA(*n* = 3). **E** HE, Ki67, and METTL9 staining in HCC-PDX tumors treated with in vivo-optimized siRNAs. Scale bars, 50 μm. **F**, **G** Quantification of Ki67+ stained cells and METTL9 staining score in tumors (*n* = 3). **H** Schematic illustration of promoting tumor growth and metastasis in HCC cells by inhibition of ferroptosis through METTL9-SLC7A11 regulation axis.

To assess whether METTL9 could be a potential therapeutic target for HCC, we utilized a patient-derived xenograft (PDX) tumor model in NSG mice (Fig. 6A). In this model, we used a specific siRNA sequence to target and silence METTL9 expression to observe its effect on tumor growth (Fig. 6B). Remarkably, silencing of METTL9 led to a significant reduction in the size and weight of PDX tumors compared to the control group (Fig. 6C, D). Additionally, the percentage of Ki67-positive cells was significantly reduced in the METTL9-silenced tumors compared to the control group (Fig. 6E–G). These findings provide promising evidence that targeting METTL9 could effectively suppress tumor growth and cell proliferation, indicating its potential as a valuable target for HCC therapy. Further research and validation in preclinical and clinical studies would be required to fully establish the efficacy and safety of METTL9-targeted therapies for HCC treatment.

## Discussion

As the first identified N1-histidine methyltransferase in mammals, it is evident that METTL9 has a carcinogenic effect on tumors. However, the detailed mechanisms of tumorigenesis and progression require further elucidation. In the present study, through comprehensive experiments in vivo and in vitro, our work was the first to report that METTL9 promoted HCC proliferation, metastasis, and invasion. Additionally, elevated METTL9 expression was associated with a relatively poor prognosis, suggesting its potential as a prognostic marker and therapy target for HCC. Mechanistically, we found that METTL9 promoted tumor progression by upregulating the expression of SLC7A11, which subsequently decreases lipid oxidative damage and enhances antioxidant capacity, thereby inhibiting tumor-cell ferroptosis. Our study provided the first evidence that METTL9 was a new ferroptosis regulator and had the property of inhibiting ferroptosis in malignant tumors.

Ferroptosis, an iron-dependent form of nonapoptotic cell death, caused by excessive accumulation of lipid peroxides due to disruption of intracellular metabolic pathways and is closely related to intracellular lipid homeostasis and iron metabolism [18, 32]. Currently, many studies have pointed out that ferroptosis was characterized by tumor-suppressing and metastasis inhibition [33, 34]. Through proteomic sequencing and KEGG enrichment pathway analysis, we found that the ferroptosis signaling pathway was enriched in datasets associated with METTL9. Hence, we undertook to validate whether ferroptosis was involved in the regulation of HCC by METTL9. Interestingly, we observed that knockdown of METTL9 increased lipid peroxidation and lipid ROS levels, indicating an enhancement of ferroptosis in HCC cells. In addition, the phenotype of METTL9 knockdown could be partially rescued by adding ferroptosis inhibitors both in vitro and in vivo. These results indicated that METTL9 promotes HCC development by inhibiting ferroptosis, which was the first evidence of METTL9’s association with ferroptosis.

Further study focused on the specific molecular mechanisms of how METTL9 regulated ferroptosis. Previous studies in the literature have shown that SLC7A11 was a key component of system Xc^-^, responsible for uptake cystine in exchange for glutamate [35], leading to the production of glutathione (GSH). GPX4 is capable of using GSH to reduce lipid hydroperoxides to lipid alcohols thereby inhibiting ferroptosis [25]. Downregulation of SLC7A11 leads to a decrease in intracellular cystine levels and subsequent reduction of glutathione biosynthesis, indirectly causing suppression of GPX4 activity and subsequently ferroptosis activation. SLC7A11 has emerged as a critical factor linking ferroptosis to its tumor suppressor function. SLC7A11 upregulation was observed in multiple tumors [36–38]. When SLC7A11 was downregulation or inactivation, genetically or pharmacologically, shown to potently induce lipid peroxidation and ferroptosis in pancreatic cancer cells, led to proliferation arrest and cell death, suggesting great potential as a strategy for anticancer therapy [39, 40]. These studies prompted us to postulate whether SLC7A11 could potentially served as a primary downstream mediator in the modulation of ferroptosis governed by METTL9. Subsequently, we validated that knockdown of METTL9 reduced the mRNA and protein levels of SLC7A11. Then, a series of rescue experiments were performed which showed that restoration of SLC7A11 in stably METTL9-depleted HCC cells partially restored METTL9 knockdown-induced suppression of cell growth, migration, and invasion. At the same time, SLC7A11 overexpression partially restored METTL9 knockdown-induced promotion of lipid peroxidation and lipid ROS levels. These results further supported that loss of METTL9 suppresses HCC progression partially through repressing SLC7A11 and promoting ferroptosis. As previously mentioned, certain studies have indicated that zinc transporter proteins might constitute substrates for METTL9 histidine methylation modifications [14]. Concurrently, other research has demonstrated that alterations in intracellular zinc ions played a role in ferroptosis regulation [41, 42]. Moreover, based on the KEGG-enriched analysis of diverse signaling pathways, METTL9 potentially exerted regulatory influence on HCC through other pathways. Consequently, we considered that SLC7A11 functions as a main downstream factor of METTL9, contributing to the regulation of ferroptosis and only partially restored the experimental results in this study. On the other hand, we postulated that METTL9, as a histidine methyltransferase, might act as a regulatory role in ferroptosis via the modification of SLC7A11’s histidine methylation. Regrettably, the unavailability of specialized reagents or antibodies tailored to histidine methylation hindered the execution of in-depth mechanistic experiments. In forthcoming endeavors, with an enhanced comprehension of METTL9 and advancements in technology, we intend to investigate deeper into this field.

Clinically, METTL9 emerged as a prognostic indicator for adverse outcomes in patients with HCC. Our experiments verified that METTL9 knockdown significantly impedes tumor growth in PDX model, highlighting its potential utility as a therapeutic target for HCC. In the future, we envisage the exploration of small molecule inhibitors or drugs as a means to further assess the viability and value of targeting METTL9 for HCC treatment.

In conclusion, our present work unveils the vital role of METTL9 in promoting HCC growth and metastasis. Moreover, we provide a novel mechanism of METTL9 mediated HCC progression by regulating ferroptosis. The findings that METTL9 facilitates evasion of ferroptosis provide a proof-of-principle, and its potential oncogenic function highlights its significance as a therapeutic target for cancer treatment.

## Materials and methods

### Reagents

Erastin (S7242), Ferrostastin (S7243), Liproxstatin (S7699) were obtained from Selleck.

### Analysis of TCGA and gene expression omnibus (GEO) databases

The pan-cancer RNA-seq data and clinical information of LIHC from The Cancer Genome Atlas (TCGA) database (https://portal.gdc.cancer.gov/) and transformed the data in TPM format. Differential gene expression analysis between tumor samples and nontumor samples was conducted by R software 4.2.1 version. Then, the standardized data were used for grade classification, pathological stage correlation analysis and survival analysis. R-package ggplot2 was used to visualize the data. R-package survminer was used for survival curve and data visualization. The two other expression data of HCC patients were downloaded from the National Center for Biotechnology Information (NCBI) GEO dataset with accession number: GSE 36376, GSE 76427.

### HCC tissue microarray

227 cases human HCC tissues and matched adjacent non-tumor liver tissues microarray from 2008 to 2013 were obtained from patients who received curative surgery at Sun Yat-sen University Cancer Center (SYSUCC, Guangzhou, China). The study complied with the standards of the Declaration of Helsinki. The Institutional Review Board of SYSUCC approved this study. (Approval No: B2023-402).

### Cell culture

MIHA (the normal liver), human HCC cell lines, Hepa1-6 and HEK293T were purchased from the Shanghai Cell Bank of the Chinese Academy of Sciences (Shanghai, China). Cells were cultured in Dulbecco’s modified Eagle’s medium (DMEM; Thermo Fisher, USA) supplemented with 10% fetal bovine serum (FBS; Gibco, California, USA) and 100 U/ml penicillin-streptomycin under standard culture conditions (37 °C, 5% CO2). All the cells were certificated with STR (short tandem repeat) and tested for mycoplasma contamination.

### Cell proliferation assays

For cell proliferation, 1.5 × 10^3^ indicated cells were seeded into 96-well plates. Cell Counting kit-8 (CCK-8, DOJINDO, Japan) was used according to the instructions. Five replicate holes were set in each group, and tested for 5 consecutive days. All experiments were performed three times.

For Edu cell proliferation assay, 8 × 10^4^cells were seeded in a 6-well plate and cultured for 24 h. Then cells were incubated with EdU reagent for 2 h and detected by BeyoClick™ EdU-594 kit according to the manufacturer’s instructions (C0078S, Beyotime, China).

For colony formation assay, 1 × 10^3^ cells were cultured in 6-well plates, about 14 days later, the cells were fixed in methanol for 15 min and stained with crystal violet for 30 min. All experiments were performed three times.

### In vitro transwell assays

For transwell assays, transwell chambers (8 μm por size; Costar) and chambers with Matrigel (Kennebunk, USA) were used to assess in vitro cell migration and invasion, respectively according to the manufacturer’s instructions. In brief, HCC cells were seeded into the upper chamber of each insert. After being incubated for the corresponding hours at 37 °C, the cells were fixed in methanol and stained with 0.1% crystal violet, then counted under the microscope.

### Cell viability and cell death assays

For cell viability, 8 × 10^3^ cells were seeded into 96-well plates. The corresponding concentration of the drug was added and incubated for 24 h, then CCK8 kit was used according to the instructions. For cell death, 3 × 10^5^ cells were seeded in 6-well plates per well. On the second day, the cells were treated with the indicated compounds, incubated for 24 h, viable and floating dead cells were collected, stained with propidium iodide and analyzed by flow cytometry. All experiments were repeated three times.

### Lentiviral transduction and generation of stable cell lines

Lentiviruses were produced by transfecting HEK293T cells with shRNA-targeting plasmids and the helper plasmids pMD2.G and psPAX2. The cell supernatants were harvested 48 h after transfection and were used to infect cells in the presence of 5 ng/ml of polybrene (Sigma). Forty-eight hours after infection, cells were subjected to 2 mg/ml puromycin selection for one week. ShRNAs were purchased from Kidan Biosciences co., Ltd (guangzhou, China), and the most effective two shRNAs were used for the experiments. The targets of METTL9 shRNAs sequence were listed in Supplementary Table 1.

### Transient transfection Small interfering RNA for SLC7A11

Small interfering RNA (siRNA) for SLC7A11 was purchased from Kidan Biosciences co., Ltd (guangzhou, China). Transient transfection siRNA was performed with Lipofectamine-RNAiMAX (Invitrogen,Carlsbad, CA) according to the instructions. Knockdown efficiency was identified by qRT-PCR and western blot 48 h and 72 h after cotransfections, respectively. Targeting sequences of SLC7A11 were listed in Supplementary Table 1.

### RNA extraction, quantitative real-time RT-PCR (qRT-PCR)

Total RNA was extracted using the RNA-Quick Purification kit (ESscience Biotech) according to the manufacturer’s instructions. cDNA synthesis was used with Color Reverse Transcription Kit (EZBioscience, USA) according to the manufacturer’s instructions. Then, the reverse transcription products were used for qRT-PCR with SYBR Green PCR kit (Invitrogen, Califonia, USA). All experiments were repeated three times. The primer sequences are listed in Supplementary Table 2.

### Lipid peroxidation and Lipid ROS assay

3 × 10^5^ cells were seeded in 6-well plates per well, the second day treated with the indicated reagent for 24 h. Then treated with either 10 μM Liperfluo (L248, DOJINDO, Japan) or BDP 581/591 C11 according to the manufacturer’s instructions (L267, DOJINDO, Japan) and incubated at 37 °C for 30 min. The cells were then washed twice with 1 mL 1 × HBSS, digested with trypsin, and suspended with 350uL HBSS and analyzed by flow cytometry.

### Western blot analysis, antibodies

Briefly, cells were lysed by RIPA buffer (P0013B, Beyotime, China). BCA protein assay kit (P0011, Beyotime, China) was used to measure protein concentration. Proteins were separated by 10% SDS-PAGE and transferred to PVDF membrane, incubated with corresponding primary antibodies: anti-α-tubulin antibody (Proteintech, 11224-1-AP, 1:1000), anti-GAPDH antibody (Proteintech,60004-1-Ig, 1:2000), anti-METTL9antibody (Proteintech, 15120-1-AP, 1:1000), anti-SLC7A11 antibody (Proteintech, 26864-1-AP, 1:1000), anti-GPX4 antibody (Proteintech, 67736-1-lg) and secondary antibodies: anti-mouse IgG (Cell Signaling Technology, 7076 S, 1:5000), anti-rabbit IgG (Cell Signaling Technology, 7074 S, 1:5000). The antibodies used for immunoblotting were the following in supplementary Table 3.

### Proteomics sequencing

The proteomics sequencing was handled by Fitgene Biotech Co., LTD (guangzhou, China). Briefly, all samples were processed by Data independent acquisition (DIA) individually to assess the proteome differences. MS1 and MS2 data were all acquired, and samples acquisition by random order. The iRT kit (Ki3002, Biognosys AG, Switzerland) was added to all of the samples to calibrate the retention time of extracted peptide peaks. The statistical analysis of the DIA dataset was performed by Spectronaut 16 (Biognosys AG, Switzerland) including data normalization and relative protein quantification.

### Immunohistochemical (IHC)

IHC assays were performed on HCC tissue microarray and xenograft tumor tissues using antibodies against METTL9 (Proteintech, 15120-1-AP, 1:200), Ki‐67 (Abcam, ab16667, 1:800) and 4HNE (Abcam, ab46545, 1:100) as described in our previous published studies [43]. Details of antibodies are shown in Supplementary Table 3.

### Tumor xenografts and lung metastasis in NSG mice

All four-week-old male NSG mice were purchased from Guangdong Medical Laboratory Center (Guangzhou, China) and fed at the Animal Experimental Center of SYSUCC. All the animal studies were performed according to the Institutional Animal Care and Use Committee at SYSUCC.

For the subcutaneous tumor xenografts model, mice were randomly divided into indicated groups (*n* = 5 per group) before injection. Hepa1-6 and Huh7 stable cells were subcutaneously injected into the right side of the posterior flank of NSG mice (Hepa1-6:2 × 10^6^ cells/mouse, Huh7:4 × 10^6^ cells/mouse) to form the subcutaneous tumor model. Tumor volume was calculated by the formula *V* = *a* × *b*^2^/2, where a and b are the tumor’s length and width, respectively. The maximum tumor size was not more than 1.5 × 1.5 cm^2^. At the experimental endpoint, tumor tissues were harvested and fixed with 4% PFA for the paraffin-embedded section.

For the lung metastasis model, NSG mice (*n* = 5 per group) were injected with stable Hepa1-6 cells (2 × 10^6^ cells/mouse) through the tail vein. The mice were sacrificed after 24 days and bilateral lung tissues were harvested and a number of metastatic nodules were assessed.

### PDX-model in NSG mice

For PDX model, surgical tumor specimens excised from the patient were pathohistologically confirmed as HCC. All surgically resectable tumors were collected in accordance with the institutional review board-approved protocols of the SYSUCC. Tumor specimens from surgically resected tumors were isolated into small fragments of tissue transplanted into NSG mice to generate PDX tumors. Tumors are harvested when they reach 1cm^2^. 10% of this tumor was then re-implanted into NSG mouse, when the tumor was thus propagated for 3-4 generations, they began to be applied to the experiment. Three NSG mice were randomly grouped and implanted with HCC-PDX. siCtrl and siMETTL9#1(20 μg siRNA per mouse equivalent), encapsulated with liposomes were injected via the intra-tumor injection every three days for 20 days. At the experimental endpoint, tumors were harvested and fixed with 4% PFA for the paraffin-embedded section.

### Statistical analysis

The statistical analyses in this work were carried out using GraphPad Prism 9. Data are presented as the mean value ± standard deviation (SD). Continuous variables were compared via two-tailed unpaired or paired Student’s *t* tests. “*p*-value < 0.05” was defined as statistically significant differences. *p < 0.05, **p < 0.01, ***P < 0.001 and ****P < 0.0001.

### Supplementary information

Supplementary Information

Original full and uncropped western blots

## Data Availability

The data that support the findings of this study are available from the corresponding author upon reasonable request.
