# Supplementary material for: METTL9-SLC7A11 axis promotes hepatocellular carcinoma progression through ferroptosis inhibition
Source: Cell Death Discov. 2023 Nov 28;9:428. doi: 10.1038/s41420-023-01723-4 (PMC10684523; doi:10.1038/s41420-023-01723-4)
Supplement: Supplementary file 1 — Supplementary Information [file 41420_2023_1723_MOESM1_ESM.docx]

**Supplementary Information**

**METTL9- SLC7A11 axis promotes hepatocellular carcinoma progression through ferroptosis inhibition**

Content:

1. Supplementary Figures and Figure Legends

2. Supplementary Tables


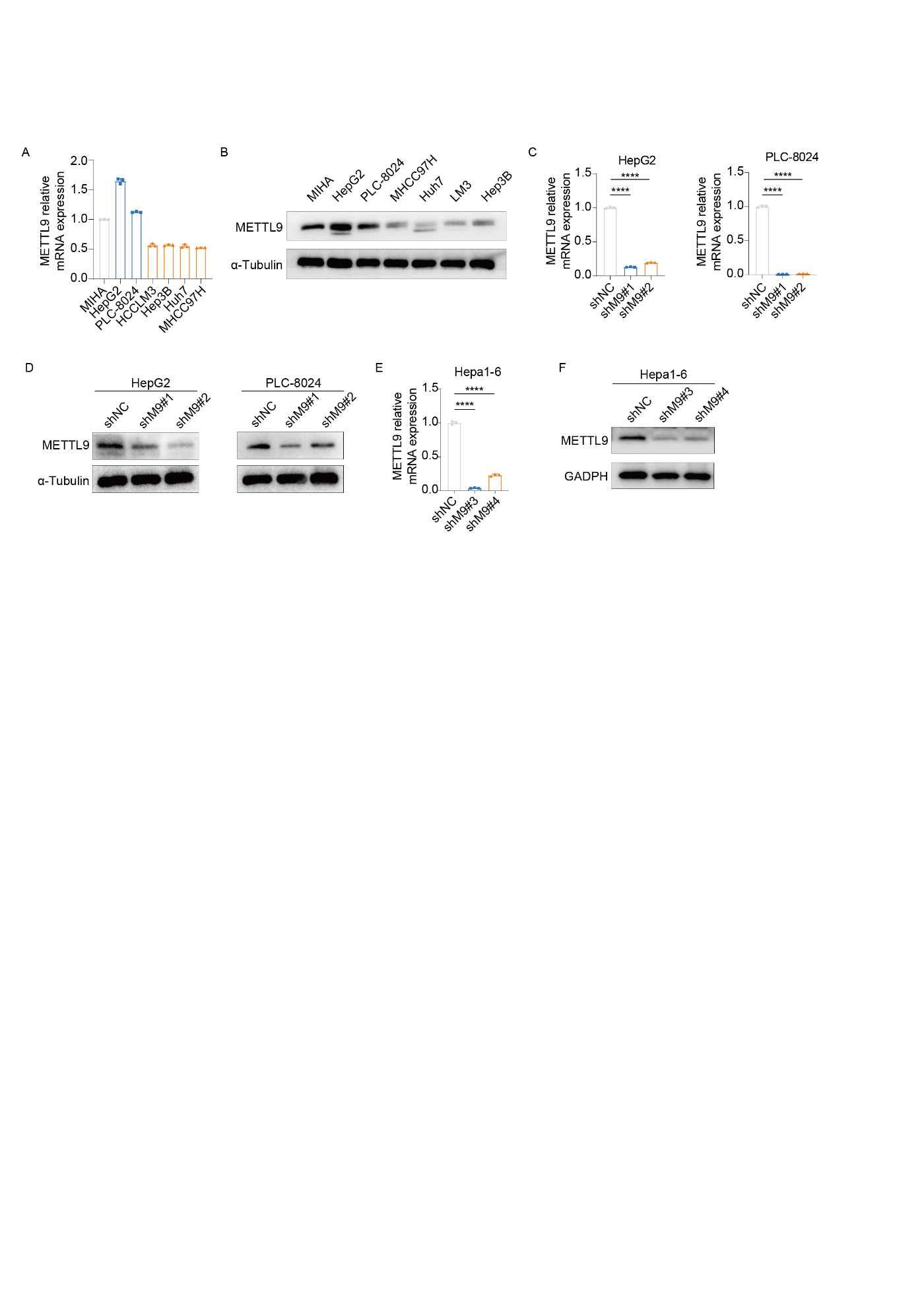


**Fig S1 | Expression of METTL9 in different HCC cell lines and efficiency verification of stable knockdown cell lines.**

**A-B** Relative mRNA and protein levels of METTL9 in different HCC cell lines and normal liver cell line MIHA were measured by real-time PCR(n=3 independent experiments) and Western blot.

**C-D** Efficiency of METTL9 stable knockdown was verified in HepG2 and PLC-8024 cell lines by real-time PCR(n=3 independent experiments) and Western blot.

**E-F** Efficiency of METTL9 knockdown was verified in Hepa1-6 cell line by real-time PCR(n=3 independent experiments) and Western blot.


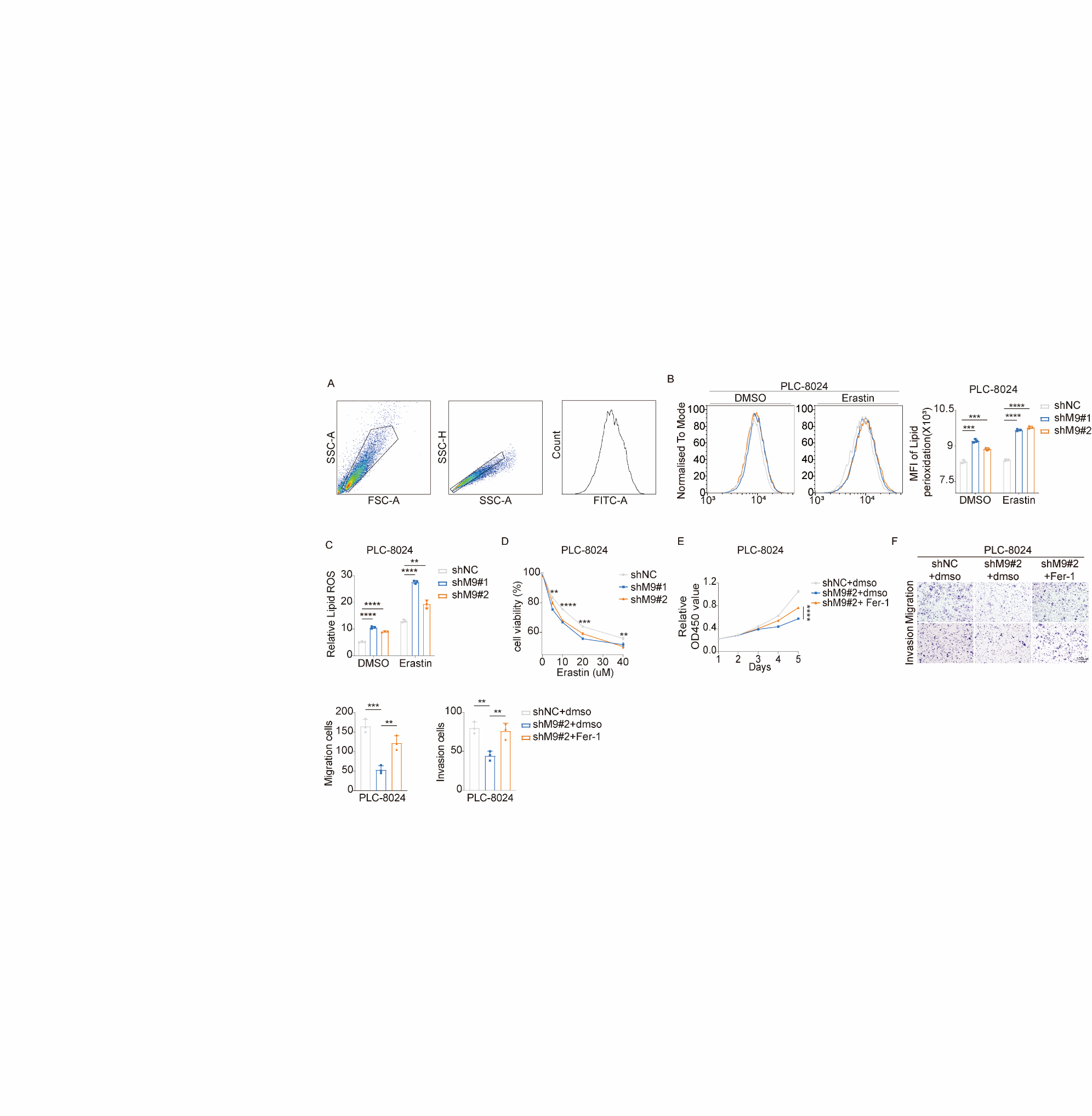


**Fig S2 | METTL9 inhibits ferroptosis in HCC.**

**A** Flowchart of loop gate in flow cytometry experiment.

**B-C** 24h after DMSO or 10uM Erastin treatment, lipid peroxidation and lipid reactive oxygen species (ROS) production were assessed respectively by Liperfluo and BDP 581/591 C11 staining in PLC-8024 stably expressing control (shNC) or METTL9 knockdown (sh#1, sh#2) cells followed by flow cytometry. n = 3 independent experiments.

**D** Cell viability of PLC-8024 knockdown cells was determined after treatment with different concentrations of Erastin for 36h by CCK-8 assay. n=3 independent experiments.

**E-F** CCK8 and transwell assays in PLC-8024 shMETTL9#2 and shNC cells with DMSO or 2 uM Fer-1. (F)Scale bar,100um.


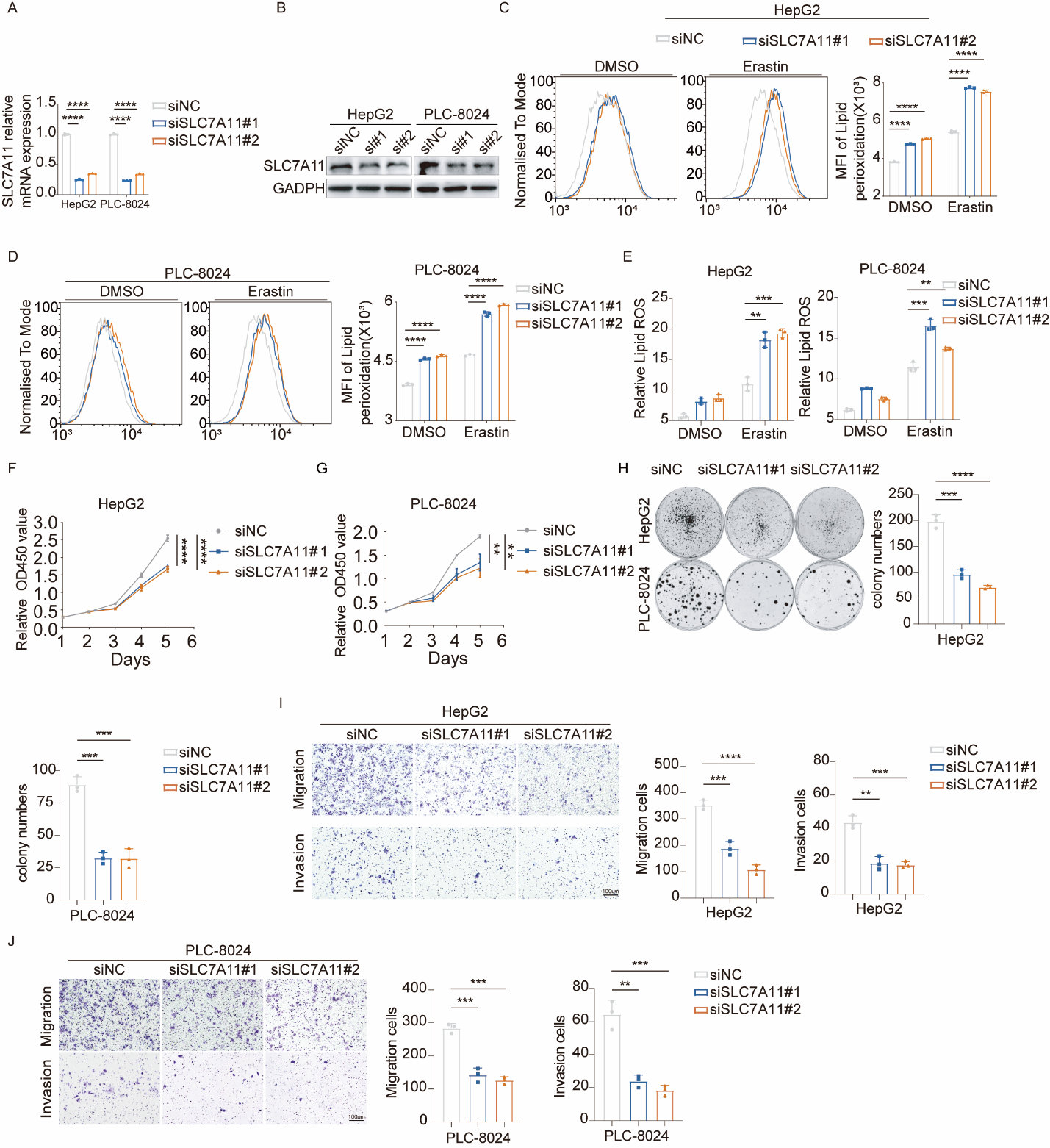


**Fig S3 | Knockdown of SLC7A11 promotes ferroptosis in vitro and inhibits HCC progression**

**A-B** Efficiency of SLC7A11 knockdown was verified in HepG2 and PLC-8024 cell lines by real-time PCR and Western blot.

**C-E** 24h after DMSO or 10uM Erastin treatment, lipid peroxidation(C) and lipid ROS production were respectively assessed by Liperfluo and BDP 581/591 C11 staining in HepG2 and PLC-8024 expressing control (siNC) or SLC7A11 knockdown (si1, si2) cells followed by flow cytometry. n = 3 independent experiments.

**F-G** Knockdown of SLC7A11 reduced cell proliferation of HepG2 and PLC-8024 cells, as evaluated by CCK-8 assay.

**H** Knockdown of SLC7A11 impaired colony formation of HepG2 and PLC-8024 cells. n=3 independent experiments.

**I-J** Knockdown of SLC7A11 significantly decreased migration and invasion of HepG2 and PLC-8024 cells by transwell assay. Quantifications of migrating and invading cells are shown as mean ± SD. n=3 independent experiments. Scale bar,100um.


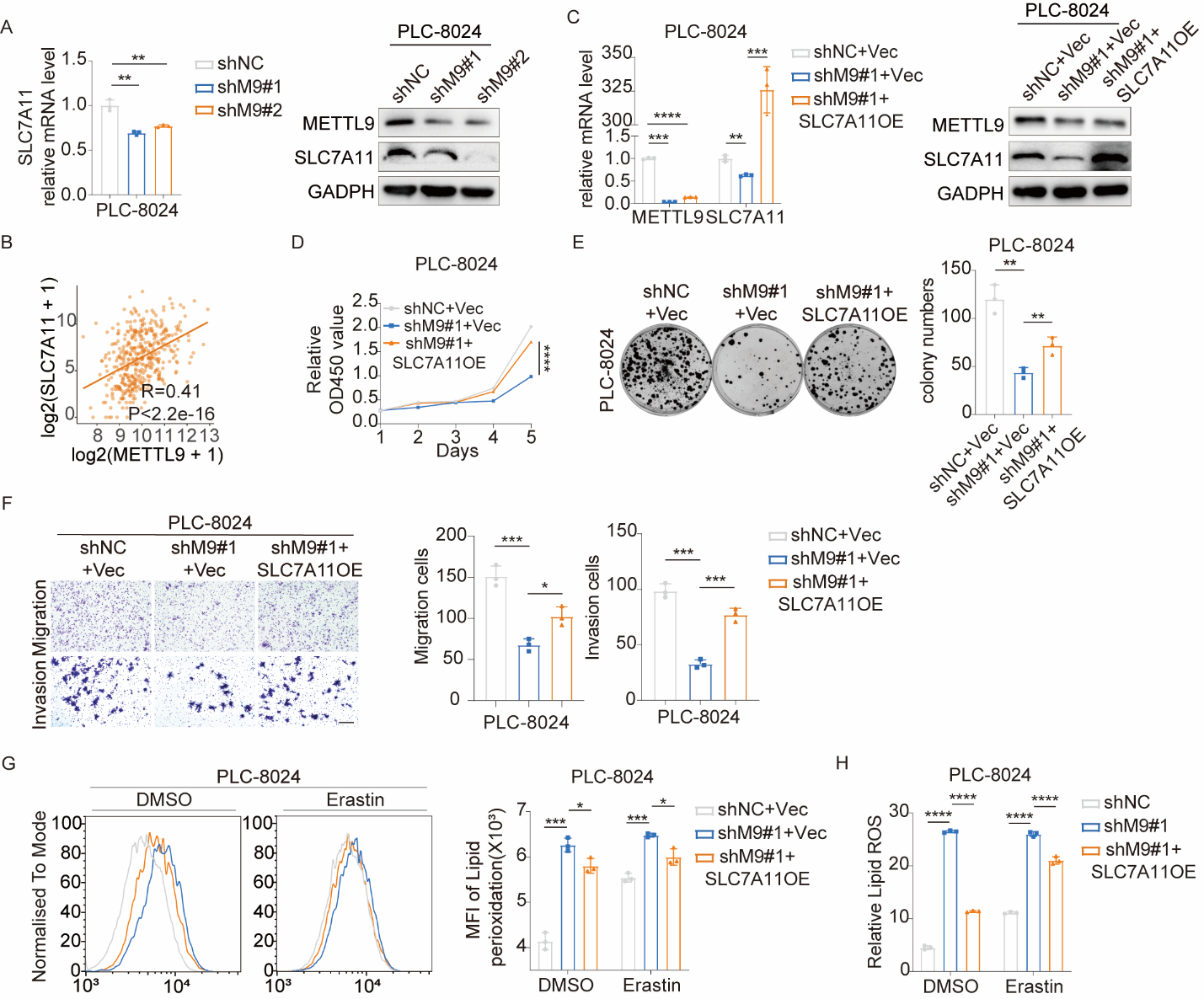


**Fig S4 | METTL9 inhibits ferroptosis in PLC-8024 cell line.**

**A** SLC7A11 relative mRNA and protein level was down-regulated in METTL9 stable knockdown PLC-8024 cell line by real-time PCR (n=3 independent experiments) and Western blot.

**B** Correlation analysis between METTL9 and SLC7A11 from TCGA-LIHC dataset.

**C** Efficiency verification in PLC-8024 cells stably expressing shMETTL9#1 with further overexpression SLC7A11 by real-time PCR (n=3 independent experiments) and Western blot.

**D-F** Cell proliferation by CCK8 assay(D) and colony formation(E) and migration and invasion assay(F) was partial rescued in PLC-8024 shMETTL9#1 cells with further overexpression SLC7A11. n=3 independent experiments. (F) Scale bar,100um.

**G-H** Lipid peroxidation(G) and lipid ROS(H) were assessed and partial rescued in PLC-8024 shMETTL9#1 cells with further overexpression SLC7A11. n=3 independent experiments.

**Supplementary Table1.**

Gene sh/si sequences used in this study.

| Gene | sh/si sequences |
| --- | --- |
| METTL9sh1 | Forward (5’-3’): GTCTTTGTTCAGAGTTACCTT  Reverse (5’-3’): AAGGTAACTCTGAACAAAGAC |
| METTL9sh2 | Forward (5’-3’): GCTGGCTATTTATCCAATTAT  Reverse (5’-3’): ATAATTGGATAAATAGCCAGC |
| mMETTL9sh3  mMETTL9sh4  SLC7A11-si1  SLC7A11-si2 | Forward (5’-3’): CGACATGTACAATGACTACTA  Reverse (5’-3’): TAGTAGTCATTGTACATGTCG  Forward (5’-3’): GACTACTATGTTCTGGACGAT  Reverse (5’-3’): ATCGTCCAGAACATAGTAGTC  Forward (5’-3’): GAAGAGGAAAGUCACUUUACUTT  Reverse (5’-3’): AGUAAAGUGACUUUCCUCUUCTT  Forward (5’-3’): GGAAGAGAUUCAAGUAUUACGTT  Reverse (5’-3’): CGUAAUACUUGAAUCUCUUCCTT |

**Supplementary Table 2.**

Primer sequences for real-time RT-PCRs used in this study.

| Gene | Primers |
| --- | --- |
| METTL9 | Forward (5’-3’): CCTGTACGTGAACATGACTAGCG  Reverse (5’-3’): AAGACAGCCTGGAGTGATTCGC |
| SLC7A11  GPX4 | Forward (5’-3’): TCCTGCTTTGGCTCCATGAACG  Reverse (5’-3’): AGAGGAGTGTGCTTGCGGACAT  Forward (5’-3’): ACAAGAACGGCTGCGTGGTGAA  Reverse (5’-3’): GCCACACACTTGTGGAGCTAGA |
| β-actin | Forward (5’-3’): CACCATTGGCAATGAGCGGTTC  Reverse (5’-3’): AGGTCTTTGCGGATGTCCACGT |

**Supplementary Table3.**

Antibodies included in the study.

| Assays | Antibodies |
| --- | --- |
| western blotting assays | Rabbit anti-METTL9 antibody (Proteintech, 15120-1-AP, 1:1000); rabbit anti-SLC7A11 antibody (Proteintech, 26864-1-AP, 1:1000); mouse anti-GPX4 antibody (Proteintech, 67736-1-lg); mouse anti-GAPDH (60004-1-Ig, Proteintech, 1:2000); rabbit anti-α-tubulin antibody (Proteintech ,11224-1-AP, 1:1000) |
| immunohistochemical staining (IHC) | Rabbit anti-METTL9 antibody (Proteintech, 15120-1-AP,  1:200); rabbit anti-Ki67 (Abcam, ab16667, 1:800); rabbit 4HNE (Abcam, ab46545, 1:100) |
