# Supplementary figures and images for: METTL9-SLC7A11 axis promotes hepatocellular carcinoma progression through ferroptosis inhibition
Source: Cell Death Discov. 2023 Nov 28;9:428. doi: 10.1038/s41420-023-01723-4 (PMC10684523; doi:10.1038/s41420-023-01723-4)

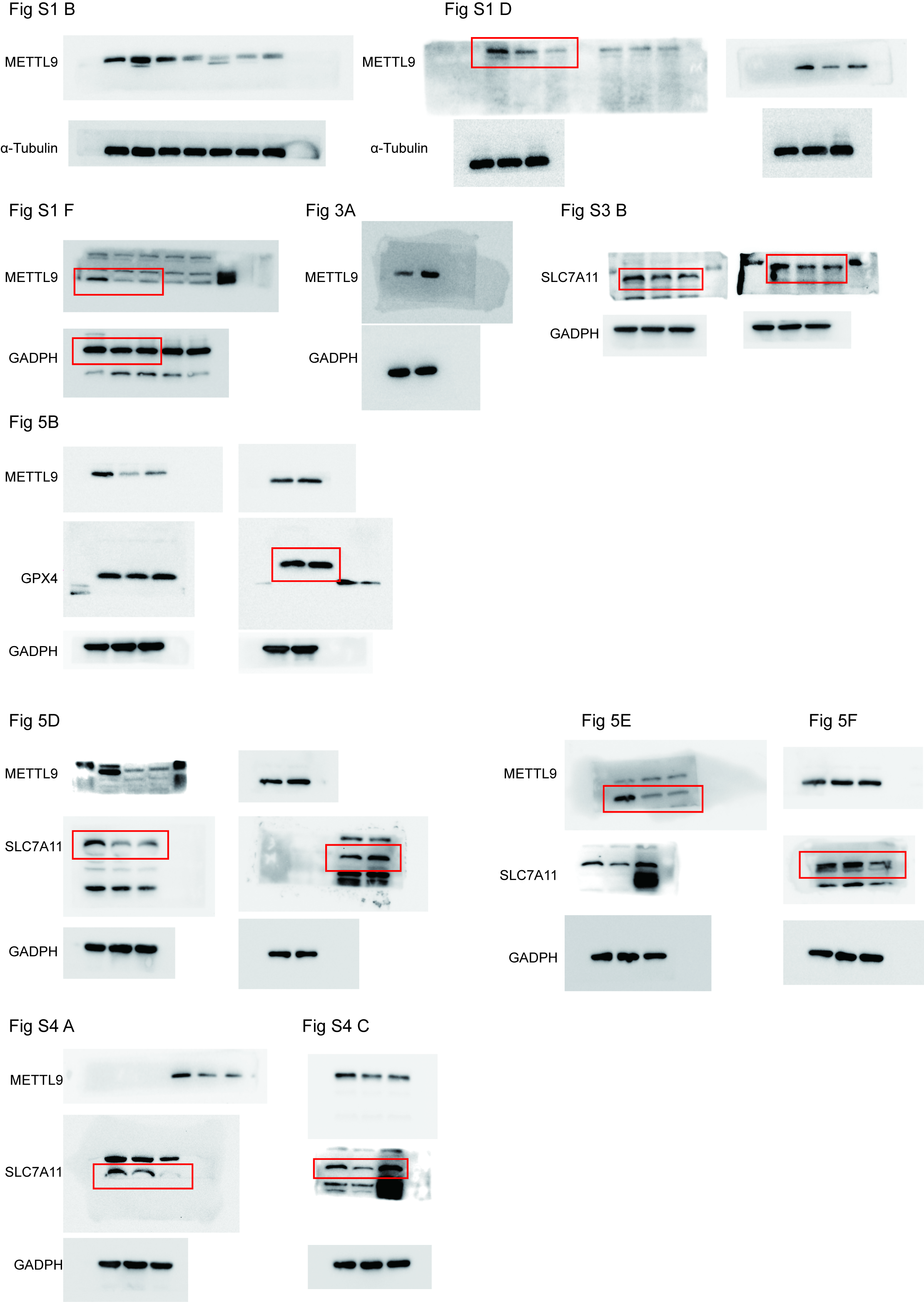

Supplement: Supplementary file 2 — Original full and uncropped western blots [file 41420_2023_1723_MOESM2_ESM.tif]
